# Shifts in Traditional Methods of Coping Among Elderly Bedouin Men

**DOI:** 10.3390/ijerph16010004

**Published:** 2018-12-20

**Authors:** Khaled Al-Said, Orna Braun-Lewensohn, Ephrat Huss

**Affiliations:** 1 Conflict Management & Resolution Program, Ben-Gurion University of the Negev, Beer-Sheva 8410501, Israel; haled70@gmail.com; 2 Department of Social Work, Ben-Gurion University of the Negev, Beer-Sheva 8410501, Israel; ehuss@bgu.ac.il

**Keywords:** Bedouins, elderly, stress, coping, culture

## Abstract

Elderly Bedouin men in southern Israel are a unique traditional population living in remote unrecognized villages and experiencing rapid social transition, in addition to deep poverty and political tension. In this study, we aimed to explore stressful events, as self-defined by the participants, and the ways in which these men have coped with those stressful events. This study involved 12 men, aged 69–74, who participated in in-depth narrative interviews during which they were asked about transformative stressful events in their lives and how they had managed, understood, and utilized human capital, meaning-making, and other methods of coping. Analysis of the interviews revealed several themes: (a) the definition of stressful events within the cultural context, (b) the use of human capital to overcome those events, (c) the transformation of experience from hindsight into a didactic narrative that can be used to assign meaning to past events, which can then be passed on to the next generation, and (d) cultural transition as a catalyst for the creation of new understandings of events. This paper sheds new light on how elderly indigenous Bedouin men self-define stressful situations within a complex and unstable cultural context. This specific context, can help us to gain insight into how indigenous impoverished older men in similar contexts may self-define their stress and coping, based on the types of generalization accepted in qualitative research. The methodological contribution of this work lies in its use of narrative to culturally contextualize phenomenological meaning structures. Its theoretical contribution lies in its examination of the concept of stress within a specific cultural context.

## 1. Introduction

Older Bedouin men are part of the last generation in their communities to have grown up in closed traditional tribal groups. Their society has undergone an intense cultural transition over the course of their adult lives, an experience that is known to be highly stressful. Over recent decades, this society has experienced rapid change from a collectivistic to a more individualistic culture and from a traditional lifestyle to more Western lifestyle. These men are in a unique position to shed light on traditional cultural norms, and how they themselves have coped with the first transitions to Western culture. This enables us to learn about these men’s lives, as well extreme cultural transitions. This work aims to explore how personal capital is defined by older Bedouin older men in Israel, in the context of their experiences of coping with stress. It also aims to see how those definitions correlate with salutogenic theory and its core components of meaningfulness, manageability, and comprehensibility, in the context of the human capital that is available in a traditional collectivist society.

Abu-Saad [1] defined traditional Bedouin society as “high context,” with an emphasis on the collective over the individual, a slow pace of societal change, and a sense of social stability as a value. Collectivism is a central component of Bedouin culture. Elsayed [2] described Bedouin culture as a culture with an outer locus of control. In terms of relationships, patriarchal power is central on the familial, institutional, and public levels. Individual actions reflect upon the family as a whole and vice versa. The values of generosity, hospitality, reciprocity, pride, valor, and strength are expressed through societal codes of indirect communication, conflict avoidance, and the use of mediators [1,3]. In this collectivist context, problems and their moral connotations are conceptualized externally within the family rather than within the psyche of the individual. Problems are solved through the mutual responsibility of the group for the individual, within the cultural norms of respect for people in authority, privacy of feelings, the importance of politeness, and belief in fate [4,5].

Patriarchal societies define the man as the center of the family and tribe, and assign older men more authority than younger men. The older man can help solve conflicts and is a center of financial social and political power in the society. The modernization and urbanization processes that the Bedouin have undergone in recent years have disrupted this framework and led to the fragmentation of the cultural hierarchy. That fragmentation has been accompanied by severe poverty, marginalization, and conflicts over land ownership, as well as unemployment, crime, and youth dropping out of school. As a result, some of the collectivist characteristics of the society have been lost and this loss is reflected in changes in traditional frameworks, the loss of traditional authority, the striving of young people for leadership and individuality [6], and the improved status of women [7]. These changes have also made it more difficult for the older generation to provide for their families financially [1,2]. This type of reduction in the power held by the traditionally powerful often leads to the intensification of control over women and to a return to traditional and religious lifestyles [4,6]. It is important to understand that the older men interviewed in this study, grew up in the traditional Bedouin culture, but have also experienced the shifts in cultural norms described below. Despite these changes, most of the Bedouin in Israel remain close to their tradition and their collectivist behavioral patterns have not completely disappeared [3]. 

## 2. Human-Capital Theory

Human-capital theory refers to the ability of actors to extract benefits from their social structures, networks, and membership in those networks [8,9]. It has been suggested that social networks provided by extended family, community, or organizational relationships may supplement the effects of education, experience, and financial capital [10,11]. Human capital is multidimensional and exists at both the individual level and the organizational level [12]. A precise link between definition and operationalization is necessary to explain any aspect of the many network processes and reciprocities characterized under this umbrella term [8]. One way of understanding how human capital is operationalized is through the phenomenology of how people understand, make meaning of, and manage their social capital. Thus, the salutogenic theory may help us to understand the role of a salutogenic sense of coherence (SOC) in the use of social capital.

## 3. The Salutogenic Model and SOC as Human Capital 

Antonovsky [13] proposed the novel concept of *salutogenesis* (origin of health) for use in the study of stress. In the salutogenic model, the health of an individual at a particular moment is seen as occupying some point on a health–disease continuum [14]. According to this model, individuals possess general resilience resources (i.e., human capital) that help them to conceptualize the world as organized and understandable. 

SOC is an enduring tendency to see the world as manageable (i.e., the individual believes that he or she has the resources needed to deal with any situation that may arise), more or less comprehensible (i.e., the internal and the external world are perceived as rational, understandable, consistent, and predictable), and meaningful (i.e., the individual is motivated to cope and committed to emotionally investing in the coping process). Antonovsky theorized that SOC plays an important role in the way one perceives challenges throughout one’s life [14].

As compared to an individual with a weak SOC, an individual with a strong SOC is less likely to perceive stressful situations as threatening [15]. It is not clear whether culture has a central moderating effect on SOC or whether individual differences in SOC mainly reflect emotional, psychological, and biological variation among individuals [16]. 

Antonovsky [14] emphasized how some cultural factors can contribute to the development of a strong SOC (see also [17]). Antonovsky [13] also argued for the importance of a stable culture for providing individuals with a strong SOC. Yet in the case of a society undergoing change, maintenance of SOC is dependent on the flexibility with which people approach and respond to new demands [14]. Antonovsky suggested that a person with a strong SOC will have the motivational and cognitive resources necessary to transform his or her other potential resources into appropriate coping strategies, which may vary by culture, but which are inherently universal in character [18]. The salutogenic model would predict that, in all cultures, SOC can potentially protect against stress [14]. In the context of more modern cross-cultural theoretical frameworks [19], the concept of universalism is less central to health and social psychology and the idea that different tools and concepts are necessary to fully understand different cultures has become widely accepted [20]. In this work, we aim to adapt the salutogenic model and its concept of human capital so that the model can be applied in a culturally appropriate manner.

## 4. Method

In accordance with the literature and our research goals, we interviewed the participants. First, we asked the elderly men about stressful events they had encountered during their lives. Second, in accordance with the theories of human capital and the salutogenic paradigm, we explored the question of which aspects of personal and human capital had aided the participants in their coping with those stressful events.

### 4.1. Research Strategy 

Since the goal of this work was to capture the constructions of the participants’ self-definitions of stress and coping in their own words and in the context of their personal, subjective social realities, the qualitative-narrative method was used [21]. This enabled us to explore how the men understood and assigned meaning to their experiences, as well as the actions they took to cope with each stressful situation. The narrative approach allowed us to look at all three elements of SOC as intertwined parts of the narrative of coping [22]. The narrative form is also well suited to the oral narrative tradition of the elders in the Bedouin community [23]. 

### 4.2. Research Population and Methods

We interviewed 12 men between the ages of 64 and 75 from unrecognized Bedouin villages in southern Israel. Each of these men participated in an open-narrative interview conducted in his home over a period of 1 to 2 h (including hosting rituals and prayer rituals that pay respect to the participant). Demographic information about the participants is presented in Table 1. The participants were interviewed by the first author, who is a well-known figure in the community.

Each man was asked to describe *“two events in your life that you define as very meaningful for you.”* From our initial explorations, we saw that the types of events described included a stressful component and so did not want to lose the meaningful element, and thus did not add the word “stressful” to the initial request. After the man described those events, he was asked questions concerning the significance of each event, what he learned from each experience, and how he had coped with each event. 

### 4.3. Analytical Strategy

The interviews were analyzed thematically in terms of areas of content. They were also analyzed narratively, in terms of central themes, forms of description, placement, length of text, etc. [24]. The findings of both of those analyses were then examined through the theoretical lens of Antonovsky’s theory of salutogenisis, in terms of coping-resource themes that corresponded to the concepts of manageability, comprehensibility, and meaning. 

### 4.4. Ethical Considerations 

The research participants were recruited using the snowball method, with each participant suggesting additional interviewees. The participants signed consent forms and the interviews were completely voluntary. Participants were assured that all identifying features would be removed. The names of the villages were also removed. The interviewer is not from the same village as any of the participants and so has no power relations within their tribes. The study was approved by the Ethics Committee of the Conflict Management and Resolution Program of Ben-Gurion University of the Negev. It is important to note, that older people often appreciate having an opportunity to tell their stories, especially to a respected member of the community such as the first author. Thus, the participants benefitted from this experience. The participants were very eager to be interviewed and to share their knowledge, as will be shown in the discussion of our findings. 

### 4.5. Validity and Reliability 

As stated, the first author is from the community and so he had a cultural understanding of the content of the interviews. The second and third authors are experts on qualitative research and salutogenic theory, respectively, and so the methodological, ethnographic, and theoretical issues were covered. The analyses were also shared with additional Bedouin researchers in peer-group research meetings, to verify the interpretations of the interviewer/first author. These analyses were performed by the three authors separately and then together, to take advantage of their different areas of expertise (see also [24]).

## 5. Results

### 5.1. Presentation of the Data

Interestingly, nearly all of the meaningful events chosen (22 out of 24) were defined as negative events, at least in the beginning of the narrative. We will now outline the level of content in the ethnographic context of the Bedouin culture.

### 5.2. The Choice of Events

The stressful events mentioned by the participants (from most to least prevalent) involved home demolitions, moving to another settlement, tension between tribes, honor killing, and polygamy and marital problems. Around half of the stressful events (11/24) involved the destruction of houses and the need to evacuate villages. This is related to the government’s policy of tearing down illegal houses and villages, as well as mandatory moves to sedentary settlements. These home demolitions create a sense of humiliation, confusion, and alienation among residents [2,25]. The house is a central element in Bedouin culture as it organizes the stability and wealth of the family and family interaction is centered around the house. Another study conducted among Bedouin living in unrecognized villages revealed that the house is a source of intimacy, security, hope, and dreams for the future. The house represents personal and family space [25,26]. 

Other events (6/24) included elements of collective violence such as fights between parts of the same tribe or families that caused rifts in the extended family. It should be noted that violence among families is a well-known social phenomenon in Bedouin Arab society associated with struggles over resources (e.g., land, social mobility) or demonstrations of power. It is also important to note that, in Bedouin society, the blood feud is associated with the value of honor and is seen as a way for avengers to attempt to restore honor and regain the trust of their tribe, so that they will not be considered cowardly and weak [7].

Other mentioned events related to polygamy (2/24), marital conflicts, and domestic violence (2/24). The polygamous arrangement among the Bedouin is a marriage relationship of one man with several women. Polygamy is a central social institution that is closely linked to the patriarchal social structure and the importance of the group and the tribe [27]. 

#### 5.2.1. Theme 1: The Form and Organization of the Narratives 

**Events related in chronological order**. Many of the participants mentioned an event from a long time ago, as their first event, and a more recent event as their second event. We noted that the earlier events were remembered more vividly:

*“It started a long time ago, in 1980. It started from a small fight between two groups of children in our tribe, and it developed into a huge feud in the tribe in which one person was killed,”* Salama said.

Ali said: *“My brother was killed a long time ago, his son was then two years old and now he’s 25, got married this year, but I remember the event as if it was yesterday.”*

This chronological order fits the needs of an oral storytelling tradition in that it helps the narrator organize and remember events. The fact that the long-term memory of elderly people is often stronger than their short-term memory [28] might also explain this observation. However, this finding could also point to the selection of a narrative according to its significance to the narrator.

**Events that had a powerful impact on the collective rather than the individual.** Salama described how the unrecognized village in which he had lived was torn down two years ago by the state: *“It happened two years ago and I will never forget it—the army and soldiers came, as if they were going to kill us all and wipe out our village.”*

Ahmed chose to mention an honor killing: *“We had to flee our village because we knew the other family would kill someone in our family after the murder.”*

The first part of the story includes a description of the intense stress of the event. Saleem stated the following in relation to an honor killing:

“There was much crying and screaming of children and women. The women escaped and it was scary and painful because it started from a small thing and escalated. The perpetuators now had to find protection and escape from their home—a nice, respected young man with small children was killed in the end—and now the killed man had to hide as well. We knew they wanted to seek revenge, and so you have to escape, you have no choice. We felt the humiliation of running away and of having someone killed in our family, but we had to run.”

#### 5.2.2. Theme 2: The Participants’ Personal and Social Capital

**Personal capital: The different components of SOC.** We examined the participants’ use of personal and human capital according to the different components of SOC. 

***Manageability***. The narratives started with a situation that the narrator felt he could not manage because of its immediacy or suddenness. The described events challenged their usual ways of managing things. As Salama said:

“It all happened suddenly. We thought that it was a very small problem. It started from the children and, suddenly, it had spread among the men. No one knows how it spread so suddenly. We thought it was controlled, and then it burst out like fire; we were all fighting.”

The participants also described an inability to decide what to do, as in the following example of land being taken away, as Salama continued:

“I had no choice, as the one responsible for the women and children and property of the family. Suddenly, they take it all away and offer you a few dunams of land (a dunam—in Turkish: dönüm—is an Ottoman unit of measure equivalent to 0.1 hectare or approx. 0.25 acres) and I had to decide, yes or no. I had no control over the situation.”

He also added:

“We weren’t sure what would happen the next day, if there would or wouldn’t be a fight. No one understood what was happening.”

Ahmed said: *“We didn’t know what would happen the next day, would they come or not? We couldn’t understand what would happen, when, and why, and so we couldn’t prepare.”*

Another form of lack of manageability appeared in the following example. It shows, through a negative example, the importance of the meaning attributed to revealing strength and manageability for the overall experience of coping with the stress. In the quote below, Ayuob describes feeling weak rather than strong:

“I chose the event of my son’s house being torn down, you know him. The event influenced me very much, until today, although in the meantime he has built another house and has children and a happy family. My house and family were ruined. My life was ruined; I felt like I couldn’t trust myself or others anymore. In that event, I came out as weak, as a person who couldn’t help his children. How will they look at me? I was weak and helpless.”

A sense of manageability was recovered through the use of personal and communal resources, as described below by Halel:

“We went to people around us and asked for their help. We turned to two families and also to the police. We kept working and asking and kept moving, and we knew what we wanted. We went to the lawyer and the family also came and offered money and support, and we froze the situation, and this gave me a feeling of having managed the situation well and of success.”

We see that the social-support systems included not only the family, but also the laws and the state, as well as individuals outside of the family. This manageability also included the passive acceptance of the help of others. According to Salama:

“The neighbors came and gave food and opened their homes. This helped us a lot; it was heartwarming.”

However, these experiences also included being in charge and managing things in a manner that led to being seen as a leader. As Halel said:

“The family started to see me differently, as the person to turn to who can help, and this gave them a feeling that I could be trusted and gradually they moved to trusting me in everything. So now I have a lot of strength thanks to how I managed this.”

The issue of polygamy also raises issues of manageability. As Ayoub told us:

“My cousin got divorced and was living at home with her parents, and my uncle asked me to marry her. It was sad that she was unmarried, so I married her as a second wife to maintain her honor and that of the family. She is the only daughter of my uncle and if she married out of the family, she would take the family’s property with her, so I agreed to marry her.”

We see from this quote that polygamy is seen as another way of gaining status and power, extending the family, and maintaining the power of the family. The narrative quoted above highlights the values of maintaining the honor and property of the extended family.

***Comprehensibility.*** The acquisition of new knowledge is an important component of comprehensibility. As Salem said:

“Now I understand that if you have the right papers, then you can prove the land is yours and no one will take it. We didn’t understand that. You need to understand exactly what papers to have with a lawyer.”

In a culture in transition, new knowledge must be acquired, in order to understand the legal aspects of a situation. This new knowledge could be learned from one’s children and integrated into the tribal knowledge, as can be seen from the words of Allean:

“I learned a lot from the older people about our tribe’s culture and what to do in different situations, but I also learned a lot from my children who received an education and who can explain to me how the laws work.”

This element of learning new ways of doing things provided the same sense of strength as power provided in the traditional culture. As Youssef described:

“After learning and understanding the laws, I feel strong again and I can explain and help others—the understanding gives me power.”

***Meaningfulness.*** The stressful events created situations, in which the narrators shifted to a stronger role and enhanced their own self-images through their handling of the situation. This included the comprehension element of identifying and utilizing resources, as wells as the management element of effectively utilizing those resources.

The ability to be a good father provided a great deal of pleasure and meaning to the men, as can be understood from Ahmed’s words:

“I know that they trust me and that I am a source of support and help to others. This is very important to me, that I’m a father who can protect his children, a father who understands the meaning of being a father, and no one can come to me with complaints.”

A new level of meaning or motivation to solve problems arose out of a commitment to the family and a desire to provide the family with a sense that something was being done. As Salama said:

“I thought that I would lose, but when I saw the children and women and the men who are no longer in the village, it gave me the strength to fight. No matter if I’d succeed or not, as long as they’d all see that I tried, that I was trying to solve the problem.”

This also includes, as before, the sense of identity that emerged from being an effective and respected problem-solver. That identity provided much personal meaning to the effort to solve problems and deal with stress. This element of meaningfulness was also considered on a religious-metaphysical level. As Suleman said:

“It’s true that everything is from Allah, but it still has to continue, for the children, for ourselves, and for the God that created us.”

An additional meaning structure is the didactic element described above, in that a father’s coping teaches his children how to cope, so that they will no longer need him. As Ayoub said,

“I did this and I don’t think everyone could have done it. I did it for my children and family. The family consults with me on everything and this puts me in a situation where I am important to them, even if they can manage without me today.”

As Saleh continued: *“It’s true that I helped them buy furniture. I asked his brother to lend him money, but my son can also take care of himself. He’s educated. I made sure he was educated and I taught them to trust themselves and each other.”*

The value of demonstrating strength was also described in the context of honor killing. As Hussen explained:

“We had no choice but to seek revenge. They killed my brother and left his children orphans. We had to kill someone; otherwise, the rest of the family-tribe would think we were weak. We would have lost our place in the family. Sometimes you have to be bad, in order to survive.”

The quote above illustrates two different (and sometimes contradictory) values: the avoidance of conflict and the demonstration of strength. It seems that conflict should be avoided if at all possible. But, if a conflict has already escalated, then one has to show strength and honor. This is described as a meaningfulness element, in that family honor is an important value, as well as a management event, in that by demonstrating strength one can manage future conflicts.

A different type of meaningfulness was raised in Salama’s narrative, in which he, as the father of a household, first perceived himself as weak and, as a result, experienced a threat to the overall coherence of the family. However, he was able to pull himself together and assign meaning to the stressful event and, in doing so, provided sense of meaning and identity for his family. As he explained:

“I learned a lot about myself. I learned that I am a leader, that I can lead. If I hadn’t intervened, things would have ended differently. When I look at these things today, I see that we used a lot of understanding and courage. … I am proud and I feel good that I managed to help my family, and they are proud of me because I helped a lot of families and saved a lot of houses from being demolished.”

**Social capital: Social support and social cohesion.** We evaluated the participants’ use of social capital, specifically their use of social support and social cohesion. As Halel said:

“I believed in myself, in my ability, and that my friends and family would help me. I believed that even the state would help me. It has rules and order, which can be used. When I worked like that, everything went well; I had a sense of security. So I did the right thing and now I can make a pilgrimage to Mecca in peace because I did the right thing.”

A Salama said: *“The young people overreact; they don’t know what they’re doing or what the results of their impulsive behavior can be. They aren’t thinking; they could humiliate all of us. It is important to act correctly, to respect the laws, to work with the people…”*

In the above narratives, we see a focus on utilizing social and national resources, modulating confronted and impulsive behaviors, and experiencing the self as capable and strong, as recourses for coping with stress. The fact that the situations were managed well was highlighted at the end of the stories. While these narrators emphasized their abilities and behaviors, they also mentioned that dialogue with other members of the community and family gave them a lot of strength and understanding regarding the way he coped with the event. As Saleem said,

“People came to me and said, ‘You did well. You acted right.’ Otherwise, there could have been many arrested, and the children and women would be the ones who would suffer. I am happy with how I acted; it was good. If a similar thing happened again, I would do the same. I did this because I believe in this way of doing things, that this is the way to behave. In this way, I prevented a war between the families.”

Salama said, *“I remember the event so well; I protected the children and women. I didn’t want anyone to get hurt and, indeed, no one got hurt, although it could have been such a situation, and I was very proud* *because I saved lives and honor, and this is what the young people should also do—look after the weak. This is their job, to protect weaker people.”*

As Ahmed said, “I managed to overcome the problem because we worked together and we believed in ourselves. The whole tribe calmed down and was proud of us and came to consult with us.”

#### 5.2.3. Theme 3: Didactic Narrative

One of the major problems facing Bedouin society is the dangerous driving of Bedouin youngsters. Ahmed said,

“I hope the young people learn from this accident; it is a message from Heaven to warn young people to drive more carefully.”

Another issue was violence among couples. The norm is that a woman cannot tell others about her husband’s violent behavior or complain. If she does so, she will be rejected by the society. As Hussen said:

“It was a long time ago, my wife went to visit her family without asking me for permission, and I saw this as rebellion and as a challenge to my authority and manliness, so when she came back I hit her and also separated from her.”

The didactic message in this story is that women must obey their husbands and that violence against wives is the result of their not obeying their husband. On the other hand, when acts of disobedience do occur, violence is considered permissible, as the honor of the husband is a primary meaning structure that needs to be maintained.

#### 5.2.4. Theme 4: New Understandings Lead to New Behaviors in a Society in Cultural Transition 

Other narratives included examples of learning new things from stressful experiences and shifts in understanding. These shifts led to additional social capital, which had not been accessible previously. 

In the context of a culture in transition [5], this shift toward seeking help from children and from outside of the tribe can be seen as the adoption of new methods of manageability that also shift the meaning of the role of the older man and the role of the tribe. This shows an adjustment to societal changes as a coping strategy taught by the elders. 

For example, Saleem said the following about efforts to help his son:

“I managed to build my son a house and get him married, but today I understand that I could have behaved differently. I could have absorbed more advice from my children and other good people and maybe I could have used a lawyer and friends that knew how to deal with these situations. I could have used the media and asked others to fight with me…”

This shift in understanding, meaning, and management can be clearly seen in Ayoub’s words below, concerning the negative effects of polygamy:

“I learned from my second marriage that I have to look after my children and wife because they are my support system. They are also people and they have feelings. I can’t just marry whenever I want because I have enough money. There are other things that need to be taken into account like their feelings.”

We also witnessed a shift in the definition of the role of the father, which now involves a more emotional role in the child’s life, as opposed to a provider of primarily material support. As we can see from Suleman’s words:

“I learned a lot from this; I became more able to listen to my kids. They are older and they need not only food and protection, but also to be a friend and brother. They need me emotionally. I now make more effort in this direction.”

This shift in values was also expressed in relation to honor killing, as described by Ahmed:

“You know what, I think we were mistaken in killing him. We should have tried to make peace with the family in other ways. We should have given up on our honor to prevent blood from being spilt. Today, things have to change, because everything is changing.”

As Halel said: *“Today there is law and you don’t have to solve things and get your rights through fighting.* *Today, I would get the advice of lawyers before fighting; there are better ways.”*

This message also endorses social change and contradicts the common assumption that the elders want to hold on to the traditional ways. 

## 6. Discussion

The aim of this study was to explore the use of human capital, personal and social, through interviews with elderly Bedouin men who still live in a relatively traditional environment. These men chose events from their past and defined stressful situations they had encountered during their lives and the ways in which different types of human capital helped them to overcome and grow from those events. We analyzed different personal and social resources through the lens of the salutogenic theory and its core components of manageability, comprehensibility, and meaningfulness. 

Many of the stressful events described in the participants’ narratives, related to the conflict between the Bedouin community and the Israeli government, over land and home demolitions carried out as part of that conflict. A previous study reported that these types of events are a major source of stress among Bedouin youth [29]. 

Interestingly, the other stressful events all related to intertribal and familial relationships. This could be a result of the fact that older men live most of their lives within the tribe. It could also be indicative of the fact, that these men have personally experienced the major transition that this society is going through [30]. This transition has led to a situation in which the traditional leadership no longer holds the substantial power it once held. In light of the subsequent power vacuum, situations can sometimes escalate in the absence of any agent that can stop that escalation [31]. 

This study focused on the personal and social resources used to deal with the various stressful situations, including situations within the nuclear and extended tribal family. Interestingly, our data show that the family and tribe provided support, but were also sources of stress. This is interesting in that it contradicts the common conception of traditional cultures as harmonious. Indeed, the central sources of stress stemmed from a desire to manage conflicts between different parts of the tribe or within families, including violence between husbands and wives. The message of these elders was very clear: Do not enter into direct conflicts that can lead to violence and, if necessary, move away, in order to prevent further violence.

The first set of themes emerged from the narrative form, that is, the way the events were presented. The form of the narratives corresponded to traditions of oral storytelling and involved the chronological organization of events, to make the narrative easier to remember [32]. This is to be expected from older Bedouin men who grew up on oral storytelling and may also be related to the relative strength of the long-term memories of the elderly. A criterion for choosing events seemed to be their impact on the collective rather than the individual, for example, the demolition of groups of houses, honor killings, conflicts between families, and events that involved the whole tribe. 

The stories were organized very clearly, with an introduction that presented the stressful event, a middle that showed how the narrator dealt with the event, including as many managerial details as possible, and, finally, a clear moral derived from the way the event was dealt with, which provided meaning and comprehension to the event. In the narratives, stressful events were characterized as sudden, unexpected, and unpredictable, which made them challenging for existing meaning, manageability, and comprehensibility structures. 

A didactic theme was intertwined with the theme of personal and social capital. This theme situates the meaning of a story in manner that allows it to serve as an opportunity to teach the younger generation, so that young listeners can learn from the experiences of their elders. This traditional storytelling format is based on the important role of older people in traditional cultures [33]. According to human- and social-capital theory, the Bedouin men acted through the described events to advance their common goals, which were mainly aimed at preserving the security and cohesion of the family and the tribe. We see from the structure of the stories that the aim of the narrators was to teach how to understand and manage stressful events. The role of the narrator is important and the story describes how well he managed the event and acted as a role model for the young people. To that end, the narrator’s actions are described in detail. This provides a sense of manageability for the narrator, but also for the community as a whole, as the narrator shows exactly how such a situation can be managed. From this success, a positive meaning emerges for the effort to manage the situation, along with a sense of achievement, increased respect from society, and enhanced self-esteem. If we define collectivist cultures as having an external locus of control, then the admiration of society for how a situation was managed and the fulfillment of the masculine responsibility to look after others become the meaning of one’s life [2]. 

The narrators also stressed that great efforts must be made to prevent direct violent conflict. To that end, wives must respect their husbands, young people must drive carefully, family conflicts must be solved through verbal negotiation with the sheik and elders, and women and children must be protected. The didactic message of the elders is very clear: Do not enter into direct conflicts that can lead to violence and, when necessary, move away to prevent further violence. However, when a violent conflict or physical danger does arise (as in most of the stressful situations described by the interviewees), then one has to show strength and regain one’s power over the situation, even if that requires the use violence, such as cultural sanctioned interpersonal or interfamilial violence. Otherwise, one’s power will be reduced and the hierarchical harmony will not be restored. This message contains two different and sometimes contradictory values: the avoidance of conflict and the demonstration of strength. Great efforts should be made to avoid conflict, but if conflict cannot be avoided, one must demonstrate strength and honor. This is described as a meaning element, in that family honor is an important value, but also as a management event, in that by demonstrating strength one can manage future conflicts. This can include polygamy, if it entails marrying someone in the tribe to protect the resources of the family or to ensure more children and enlarge the tribe, as opposed to losing women and children from the tribe.

As a result of the narrators’ management of stressful situations in a manner that enabled an experience of meaningfulness, the reported situations led to the creation of a sense of strength, social status, and a positive identity as one who helps others, as well as shifts in meaning structures and new understandings. Indeed, one of the most interesting findings was that although the old men symbolize the traditional culture in their communities, they have adapted to the transitional cultural context by adjusting their understandings of situations in light of that cultural transition, thereby adjusting meaning and manageability as well. We saw encouragement to comply with and work with the Israeli legal system, to fight the confiscation and demolition of houses. We saw regret at the emotional pain that polygamy created for a first family and we also saw a shift in the definition of the role of the father from physical protector to physical and emotional protector. We saw regret over honor killing, as well as efforts to find peaceful solutions that would not be disruptive of the social hierarchy/social stability. These observations point to flexibility and learning within the seemingly rigid traditional cultural worldview. In contrast to many of the findings recorded, in the existing literature, in this work, the major conflict did not appear to be between the older and younger generation, as the elders were constantly renegotiating manageability, meaning, and comprehension, in a manner that demonstrates flexibility to the youth. 

A limitation of this work is the tension between the different theories and the participants’ self-definitions. However, that is also the strength of this work, as it enables us to draw a connection between the phenomenology of salutogenic coping and the concept of human capital within a specific social context. Further research might include quantitative exploration of the relationship between these two concepts in other cultures in which human capital is both very strong within the community and lacking in relation to the hegemonic community. Further studies might also examine whether these findings remain constant among different generations, that is, among the sons and grandsons of the men interviewed in this work.

Beyond giving voice to an understudied and marginalized group in society and older men, in general, the analysis of the significant events reported by the participants in this study demonstrates that the human capital of elderly Bedouin men strengthens the cohesion of their tribes and communities and comes from a sense of belonging, commitment, and sense of meaning. The older Bedouin men who had the best management skills and were best able to take an active part in managing their lives and family life were involved, organized, and dominant participants in the decision-making processes of the community. They gained comprehension by distinguishing between conflicts that could be prevented and those that could not be prevented. The social respect they gained as a result of their effective management of a conflict led to a sense of satisfaction. Their human capital helped them to improve their quality of life and the lives of their families and to enhance their self-confidence. Indeed, the motivation for telling a story is to teach the next generation, a motivation that is intimately linked to a sense of human capital. These ideas are reflected through the different themes that were raised regarding the components of SOC (i.e., manageability, comprehensibility, and meaningfulness) and the social resources of social support and social cohesion. 

## Figures and Tables

**Table 1:** Demographic Description of the Participants.

No.	Pseudonym	Number of Children	Age	Marital Status	Number of Wives	Education	Remarks
1	Youssef	13	70	Married	2	No formal education	One of his wives passed away.
2	Suleman	10	72	Married	2	Elementary school	
3	Ayuob	14	69	Married	2	Graduated from 12th grade	
4	Salama	20	74	Married	2	*Kuttab*	One of his wives passed away.
5	Allean	22	72	Married	3	No formal education	He divorced one of his wives and now has three
6	Ali	20	63	Married	2	No formal education	
7	Saleh	18	70	Married	2	*Kuttab*	
8	Saleem	23	74	Married	2	*Kuttab*	
9	Mostafa	24	71	Married	2	No formal education	
10	Ahmed	25	74	Married	3	No formal education	
11	Hussen	19	73	Married	2	No formal education	
12	Halel	12	72	Married	4	*Kuttab*	

Note. A *kuttab* is a traditional elementary school in which reading, writing, and mathematics are taught to children of different ages in one classroom.
